# Electrical storm in a middle-aged man

**DOI:** 10.4314/gmj.v57i2.11

**Published:** 2023-06

**Authors:** Dzifa Ahadzi, Francis Agyekum, Alfred Doku, Abdul-Subulr Yakubu, Gwendolyn Hoedofia, Harold Ayetey

**Affiliations:** 1 Department of Internal Medicine, Tamale Teaching Hospital, Tamale, Ghana; 2 Department of Medicine, University of Ghana Medical School, Korle-Bu Teaching Hospital, Accra, Ghana; 3 Department of Medicine, Korle-Bu Teaching Hospital, Accra, Ghana; 4 Department of Internal Medicine and Therapeutics, University of Cape Coast School of Medical Sciences, Cape Coast, Ghana

**Keywords:** Electrical storm, ventricular tachycardia, arrhythmogenic right ventricular cardiomyopathy, arrhythmia, ventricular fibrillation

## Abstract

Electrical storm (ES) refers to a life-threatening condition characterised by three or more episodes of ventricular tachycardia (VT), ventricular fibrillation (VF), or appropriate implantable cardioverter defibrillator (ICD) shocks in 24 hours. We report a case of a 58-year-old man who suffered recurrent episodes of sustained VT despite appropriate defibrillation and antiarrhythmic drug therapy. On stepwise evaluation, arrhythmogenic right ventricular cardiomyopathy (ARVC) was considered the most likely substrate for his dysrhythmia. He was managed conservatively on antiarrhythmic drugs with no further clinical episodes of VT, and ICD implantation for secondary prophylaxis was recommended.

**Funding:**

None declared

## Introduction

An electrical storm (ES) is a life-threatening medical emergency with repeated episodes of sustained ventricular arrhythmias within 24 hours. 1 The incidence of ES in Sub-Saharan Africa (SSA) has not been well documented as ventricular arrhythmias are often under-recognized and undertreated in this sub-region.^2^ However, in Western countries, an incidence of 4% to 40% has been reported in patients with implantable cardioverter defibrillators (ICDs), with a high mortality rate of up to 14% within 48 hours.^1^There is no clear consensus diagnostic criteria. However, it is generally agreed that ES can be diagnosed based on the occurrence of three or more episodes of sustained ventricular tachycardia (VT), ventricular fibrillation (VF), or appropriate shocks by an implantable cardioverter defibrillator (ICD) within 24 hours.^1^,^3^Electrical storm (ES) may also be diagnosed if incessant VT or VF recurs after initial defibrillation or when conventional interventions fail to abort the arrhythmia.^1^

Given the high mortality rate, a prompt diagnosis is imperative, followed by appropriate emergent care. Most patients present with hemodynamic compromise or cardiac arrest preceded by a history of palpitations, light-headedness, syncope or dyspnea. 3

The initial goal is to restore sinus rhythm and hemodynamic stability. Subsequently, searching for the underlying cause is important to guide acute and long-term care. Arrhythmia care in SSA is hampered by many factors, including poor knowledge of electrocardiographic tracings that represent life-threatening arrhythmias. 2,4 This leads to undesirable outcomes in most cases 2,4 We present a case of a middle-aged Ghanaian man diagnosed with ES based on his clinical presentation and ECG rhythm, highlighting the approach to diagnosing and managing this arrhythmic emergency. A search for the underlying cause in our patient revealed undiagnosed structural heart disease as the substrate; the patient met diagnostic criteria for ARVC, a rare cardiomyopathy in individuals of African descent.^5^

## Case Report

A 58-year-old male who was previously well reported a one-month history of intermittent left-sided chest pain. The first episode occurred during his usual farm work. It was sudden in onset, sharp, non-radiating and subsided with rest. Subsequent episodes occurred spontaneously but were aggravated with activity and relieved with rest. Three days before presentation, he had a similar episode of chest pain. Two days later, he observed associated palpitations and light-headedness culminating in an episode of syncope. He was thus rushed to a peripheral facility from where he was referred after initial care for cardiac arrhythmia. On further questioning, he reported “seizures” a month prior, for which he had been started on anticonvulsant therapy. He had a remote history of asthma but no history of cardiovascular disease. His family history was remarkable for hypertension and diabetes in his father. There was, however, no known history of structural heart disease, sudden cardiac death or premature cardiovascular disease. The patient had been started on oral warfarin 5 mg daily, oral furosemide 40mg daily, oral soluble aspirin 75mg daily, oral levetiracetam 500mg twice daily and oral digoxin 250mcg daily. He was a farmer and drank alcohol occasionally (less than 14 units/week) but did not smoke tobacco or use any recreational drugs.

**Figure 1:**
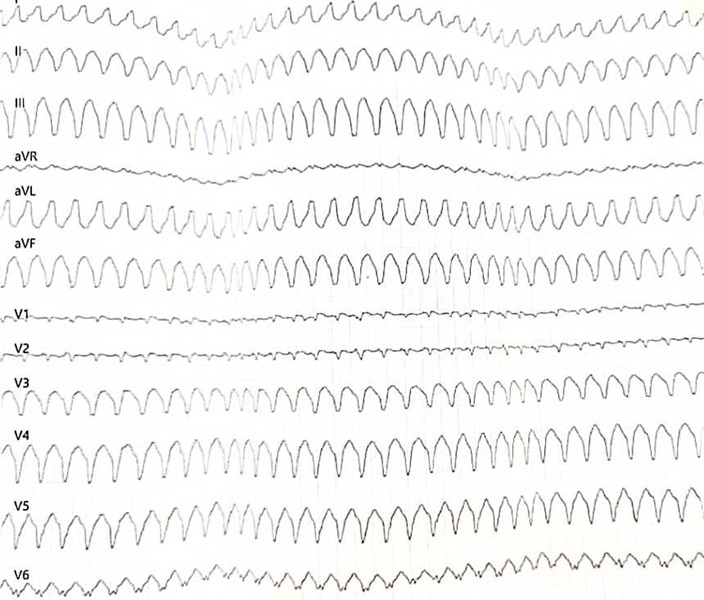
Monomorphic VT at a rate of 300bpm

On examination, the patient was a well-built man with no clinical evidence of fluid congestion. The skin was normal with no palmoplantar keratoderma. He had a regular pulse of 90 beats per minute (bpm) and a blood pressure of 144/97mmHg. The rest of the physical examination was normal. His referral ECG showed a monomorphic VT with a rate of 300 bpm (Figure 1).

**Figure 2:**
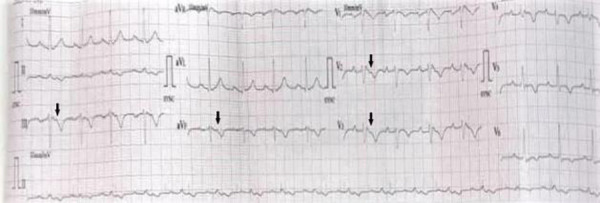
ECG on arrival at the emergency room. Incomplete right bundle branch block (RBBB), Brugada-like pattern in leads V1-V3, with widespread repolarisation abnormalities. Probable Epsilon wave in leads V2, V3, aVF and III (black arrows)

However, his ECG on presentation showed an incomplete right bundle branch block pattern, a Brugada-like pattern in leads V1-V3, and widespread repolarisation abnormalities. An additional notch akin to an Epsilon wave was observed in leads III, V1 – V3, after the QRS complex and before the T wave (Figure 2).

Differential diagnoses for the underlying cause of his monomorphic VT were a non-ST elevation acute myocardial infarction, pulmonary embolism, post-viral myocarditis, electrolyte imbalances, undiagnosed structural heart disease or channelopathy, and he was thus evaluated accordingly.

He was initiated on double antiplatelet therapy (soluble aspirin and clopidogrel 300mg stat, then 75mg daily), therapeutic anticoagulation (subcutaneous enoxaparin 80mg twice daily) and a high-intensity statin (atorvastatin 40mg daily).

**Figure 3:**
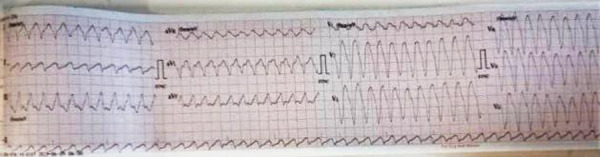
Monomorphic VT at 300 beats per minute, left-bundle branch block (LBBB) morphology with the inferior axis

The patient became hemodynamically unstable within 24 hours of admission. His ECG showed a monomorphic VT with a different morphology from the pre-admission VT (Figure 3).

**Figure 4:**
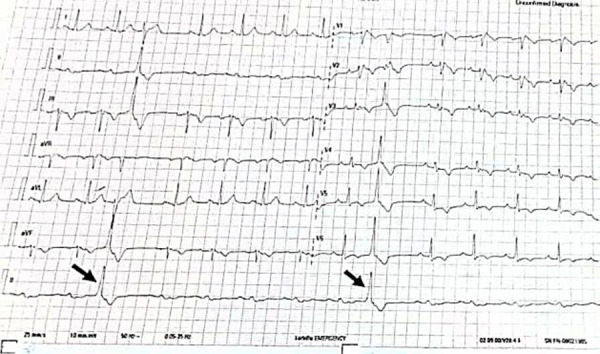
Post-cardioversion ECG showing premature ventricular complexes (black arrows) of RBBB morphology and inferior axis

**Figure 5:**
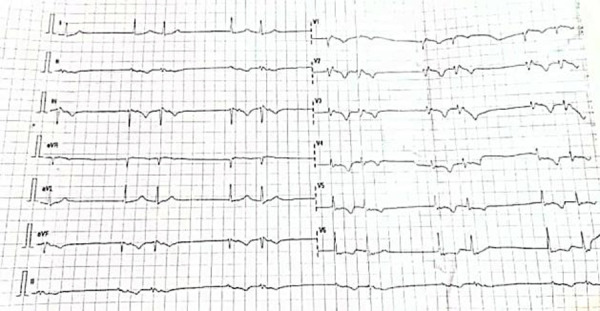
Peri-cardioversion ECG showing bradycardia with a junctional bigeminy

He was successfully cardioverted using a biphasic defibrillator at a shock energy of 120 Joules. Intravenous amiodarone was initiated (150mg in 100mls of 5% dextrose over 10mins, then 1mg per minute over 6 hours, then 0.5mg per minute over 12 hours). Peri-cardioversion, he had frequent premature ventricular complexes (Figure 4) with subsequent bradycardia (Figure 5) and recurrent episodes of hypotension.

A bedside echocardiogram revealed mild left ventricular (LV) systolic dysfunction (ejection fraction (EF) = 45%) and dilated right-sided chambers with no regional wall motion abnormalities. Preliminary blood work-up showed mild leucocytosis with differential neutrophilia and biochemical evidence of end-organ hypoperfusion (mild renal impairment, hepatic transaminitis and mild proteinuria with granular casts on urinalysis). His serum electrolytes and thyroid function tests were normal.

**Figure 6:**
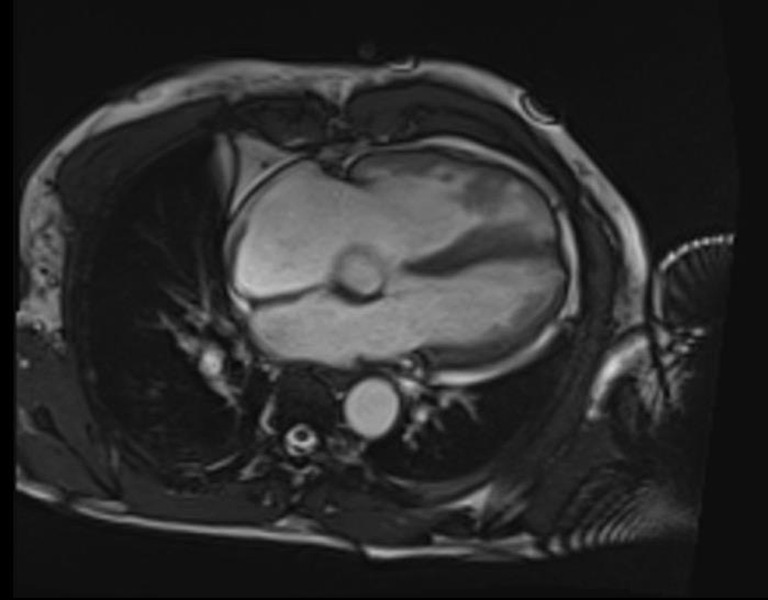
Four-chamber steady-state free procession end-diastolic bright blood cardiac MRI image showing mildly dilated RV and irregular RV wall

The patient was transferred to the ICU, where he had two additional episodes of sustained VT and an episode of cardiac arrest with the return of spontaneous circulation after 3 minutes of cardiopulmonary resuscitation. Fontaine lead placement accentuated the suspected Epsilon waves.^6^ A cardiac MRI showed irregular right ventricular (RV) walls, mildly increased RV volumes with a reduced RVEF of 45% and two RV thrombi (Figure 6). The left heart was, however, structurally normal, with an LVEF of 65%. The diagnosis of arrhythmogenic right ventricular cardiomyopathy (ARVC) as the substrate for the ES was made based on the patient satisfying the revised 2010 ARVC task force criteria.^7^ The patient had two major criteria (T-wave inversions in leads V1-3 or beyond in the absence of complete right bundle branch block and epsilon waves) and one minor criterion (sustained VT of right ventricular outflow tract configuration: left bundle branch block morphology with inferior axis) consistent with a definite diagnosis of ARVC. The primary diagnosis was Electrical Storm (ES) secondary to ARVC with right ventricular intracardiac thrombi. The secondary diagnosis was Acute Kidney Injury/Ischemic Hepatitis secondary to recurrent episodes of hypotension.

He was managed with oral amiodarone 400mg twice daily for two weeks, then 200mg twice daily, bisoprolol 1.25mg daily, ramipril 1.25mg daily and rivaroxaban 15mg twice daily for 21 days, then 20mg daily (total of 6 months). The patient was counselled on his diagnosis, emphasising the need for exercise avoidance, screening of at-risk family members and an ICD for secondary prophylaxis. He declined the ICD because of cost and opted to be managed conservatively. On follow-up, two weeks post-discharge, his liver function improved whilst his renal function remained stable (Table 1). He has since been reviewed on an outpatient basis at 3-monthly intervals. He has remained free of arrhythmias on drug therapy. Of six first-degree relatives screened, the patient's 26-year-old son had dilated right-sided chambers on echocardiography though he had no concerning symptoms and a normal 12-lead ECG. A cardiac MRI has been requested for further evaluation.

**Table 1:** Results of laboratory investigations

Variable	Baseline	Repeat	Reference range
**Renal Function**			
**Sodium, mmol/l**	134.0	135.0	135.0-150.0
**Potassium, mmol/l**	6.2	4.9	3.5-5.5
**Chloride, mmol/l**	99	101	95.0-110.0
**Urea, mmol/l**	6.4	7.8	2.0-7.0
**Creatinine,**	165	166	71.0-133.0
**eGFR (ml/min/1.73m^2^)**	48	48	>89.0
**Liver Function Test**			
**Total Bilirubin, μmol/l**	19.4	16.0	3.0-22.0
**Direct Bilirubin, μmol/l**	10.2	4.0	0.0-5.0
**Aspartate transaminase, U/L**	130.0	17.0	10.0-35.0
**Alanine transaminase, U/L**	163.0	8.0	10.0-45.0
**Alkaline phosphatase, U/L**	130.0	99.0	38.0-126.0
**Gamma-glutamyl transferase, U/L**	649.0	42.0	12.0-58.0
**Total protein, g/dl**	77.0	64.0	63.0-82.0
**Albumin, g/dl**	42.0	48.0	35.0-50.0

## Discussion

An electrical storm (ES) is a life-threatening emergency requiring prompt medical or cardiac intervention to prevent mortality.^1^ The pathophysiology of ES involves an inherent or acquired predisposition to cardiac electrical instability (substrate of myocardial scar or arrhythmic syndromes), acute triggers, and autonomic dysfunction (sympathetic overdrive or parasympathetic suppression).^3^,^8^,^9^Reversible triggers associated with ES include myocardial ischemia, electrolyte abnormalities, sepsis, decompensated heart failure, infections or fever, hyperthyroidism and antiarrhythmic drugs.^8^ The complex interplay between these factors and the fact that a trigger may not be identifiable in most cases makes management challenging and outcomes generally poor.^1^,^8^,^10^

ES due to VT is common and often occurs in individuals with structural heart disease, as seen in our patient.^3^,^8^ES may present with palpitations, difficulty breathing, chest pain and syncope.^1^,^4^Sudden hemodynamic collapse, cardiac arrest and repeated episodes of dangerous arrhythmias may be the initial presentation in high-risk patients.^1^In such cases, emergent resuscitation is the initial goal of care.^1^ES should be managed in intensive care or high-dependency unit settings where patients can be closely monitored. Advanced care was provided as necessary. 8 Clinical evaluation should be comprehensive, including a detailed history and clinical examination, focusing on identifying and treating reversible triggers and revealing an underlying substrate.^3^ Implantable cardioverter defibrillators should be interrogated and reprogrammed as appropriate.^8^

Medical therapy is important in reducing the recurrence of ES but has little impact on mortality.^9^ES is managed with non-selective beta-blockers, such as propranolol, which suppress sympathetic overdrive.^9^In our patient, a cardio-selective beta-blocker was prescribed due to concern of precipitating his asthma, with good results. Amiodarone has multi-channel blocking properties, making it one of the most effective antiarrhythmic drugs in the management of ES. 9 Further therapeutic options for refractory ES include deep sedation, mechanical ventilation, catheter ablation, and autonomic modulation (stellate ganglion block and thoracic epidural anaesthesia).^9^

A poor synthesis of our patient's clinical features and ECG rhythm may have accounted for the misdiagnosis of his arrhythmia and subsequent choice of therapy before referral to our facility.

This highlights the need for basic ECG and arrhythmia care training for primary care providers, as they are often the first point of contact for these patients.

After ES has abated, secondary prophylaxis with an ICD is indicated in most patients. Despite discussing this with our patient, he declined due to the high cost. Arrhythmia management in SSA is typically non-invasive due to the high cost of device therapy, lack of national health insurance schemes that cover the cost, and scarcity of cardiac personnel, including electrophysiologists in the sub-region. 11 Most cardiac devices implanted in SSA are done on a humanitarian basis by foreign donors, occasionally with recycled devices,^11^hence device therapy is largely unavailable as a therapeutic option for many patients in SSA.

The substrate for ES in this patient was arrhythmogenic right ventricular cardiomyopathy (ARVC) based on the revised 2010 ARVC task force criteria.^7^ The prevalence of ARVC is reported as 1 in 1000 to 5000 persons in the West^11^ with limited data in SSA (50 cases in 5 years in South Africa)^2^. The disease is characterised by fibrofatty replacement of the myocardium^12^ and commonly affects the right ventricle, as seen in this case, but may affect both ventricles or the left ventricle in isolation.^7^It is often inherited in an autosomal dominant fashion. However, autosomal recessive inheritance can occur.^13^ Symptomatic patients have an increased risk of sudden cardiac death and should be stratified with ICD implantation considerations.^13^ Our patient was considered high risk given the occurrence of ES as the initial manifestation of his disease. However, ICD implantation was mitigated by cost. Avoidance of exercise is a key strategy in preventing further episodes of ES in patients with ARVC.^8^ Also, serial screening of at-risk family members is mandatory as the disease is heritable.^8^

**Ethical Considerations:** The patient provided informed consent to publish this case.

## Conclusion

An electrical storm (ES) is a life-threatening medical emergency that often presents as sustained VT with haemodynamic instability or cardiac arrest, as seen in our patient. Patient survival depends upon promptly recognising the arrhythmia and instituting emergent care as appropriate. This case highlights the challenges of arrhythmia care in our sub-region and the need to make life-saving device therapy affordable and accessible.
